# Primary aortoduodenal fistula presenting as a rare cause of upper gastrointestinal bleeding

**DOI:** 10.1002/ccr3.6888

**Published:** 2023-01-16

**Authors:** Haitham Rejab, Sami Fendri, Ahmed Bouzid, Ayman Dammak, Hela Ben Jemaa, Ayman Trigui, Salah Boujelben

**Affiliations:** ^1^ General Surgery Department Habib Bourguiba Hospital, University of Sfax Sfax Tunisia; ^2^ Cardio Vascular Surgery Department, Habib Bourguiba Hospital University of Sfax Sfax Tunisia

**Keywords:** aortic aneurysm, aortoenteric fistula, primary aortoduodenal fistula

## Abstract

Primary aortoduodenal fistula (ADF) is a serious life‐threatening condition. Unlike secondary ADF which occurs in patients who had previous aortic prosthetic reconstruction, primary ADF is uncommon. Its diagnosis is often unsuspected until surgery or postmortem. We report a rare case of primary ADF presenting as massive gastrointestinal bleeding.

## Graphical abstract

Primary ADF is a life‐threatening complication that should be considered in all patients with known abdominal aortic aneurysms presenting with massive bleeding. Early diagnosis and emergent surgery are crucial to patient survival.

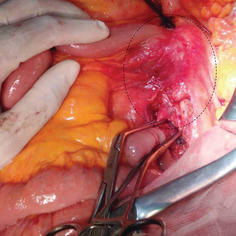

## 1 CASE PRESENTATION

**FIGURE 1:**
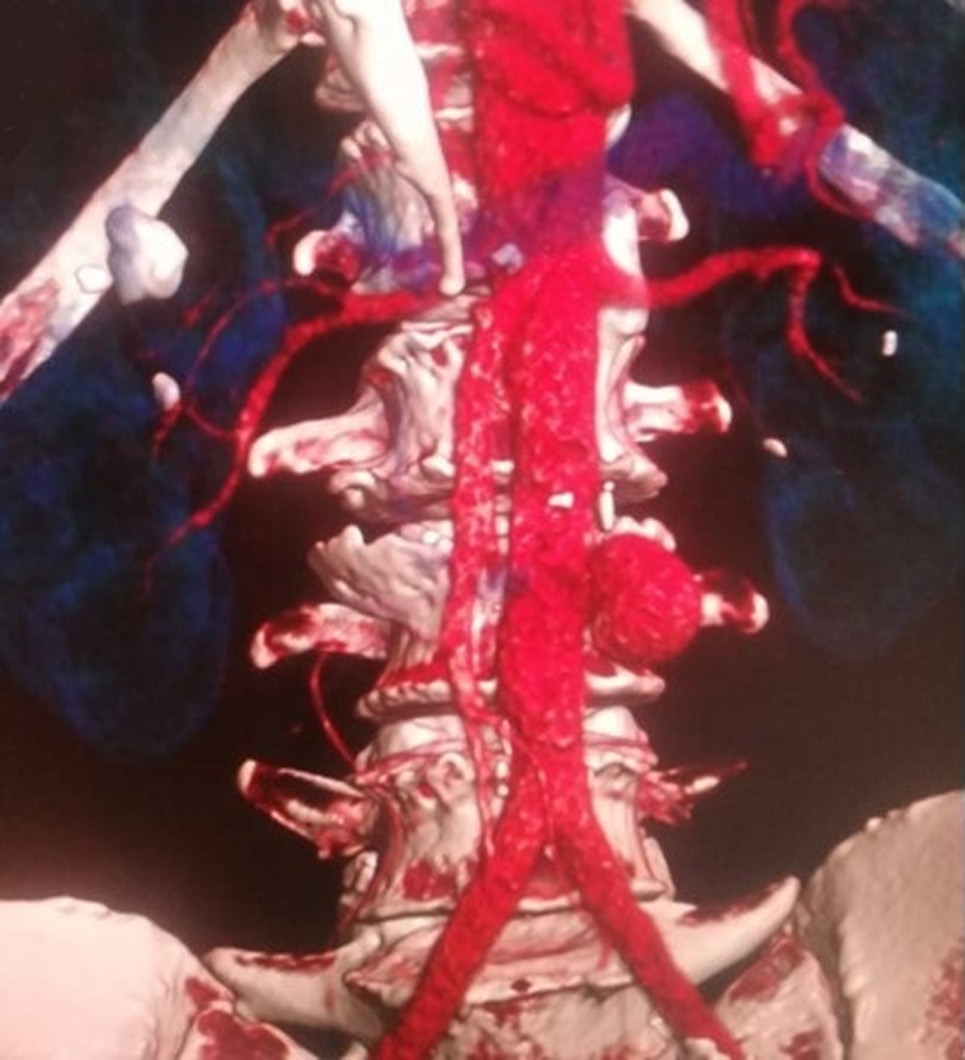
3D reconstruction of the abdominal CT scan showed an infra‐renal abdominal aortic aneurysm

**FIGURE 2:**
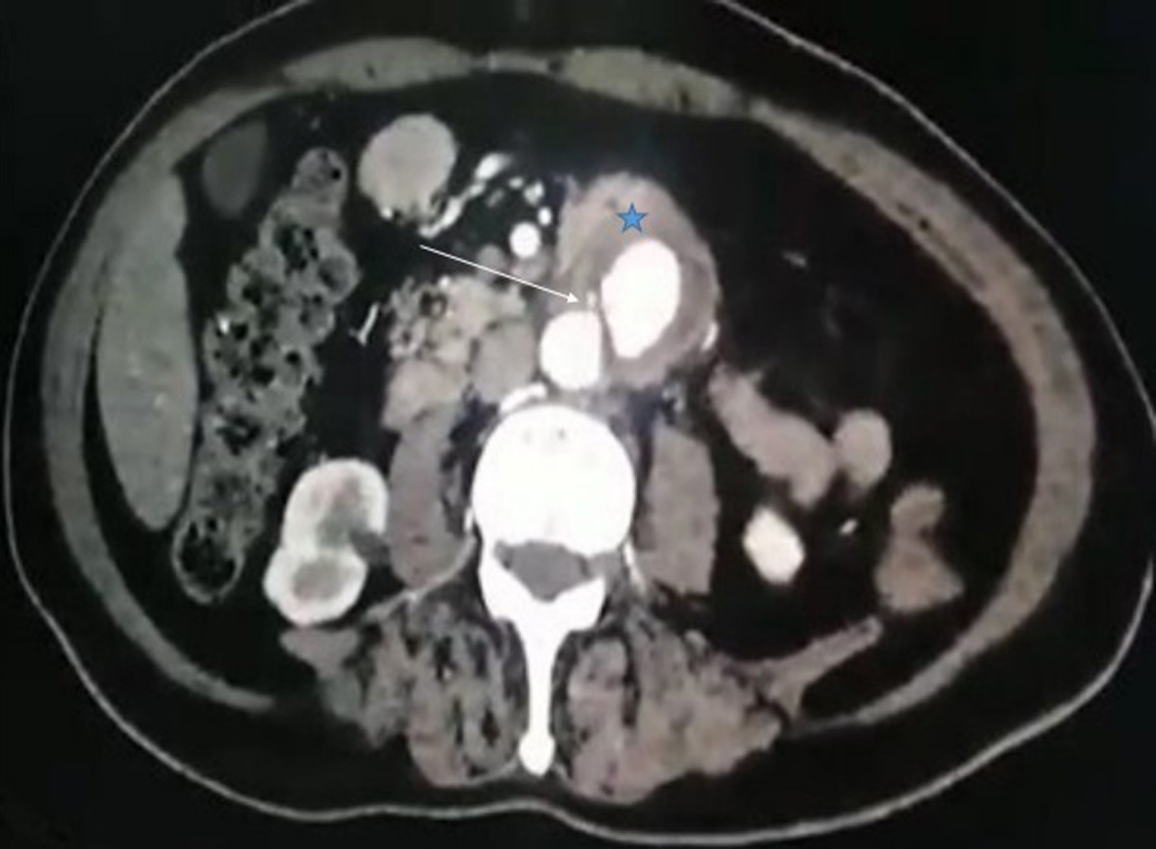
Axial CT scan revealing induration around the aorta (blue star) with an adjacent fat strand abutting the small bowel and contrast leakage toward the duodenum (white star).

**FIGURE 3:**
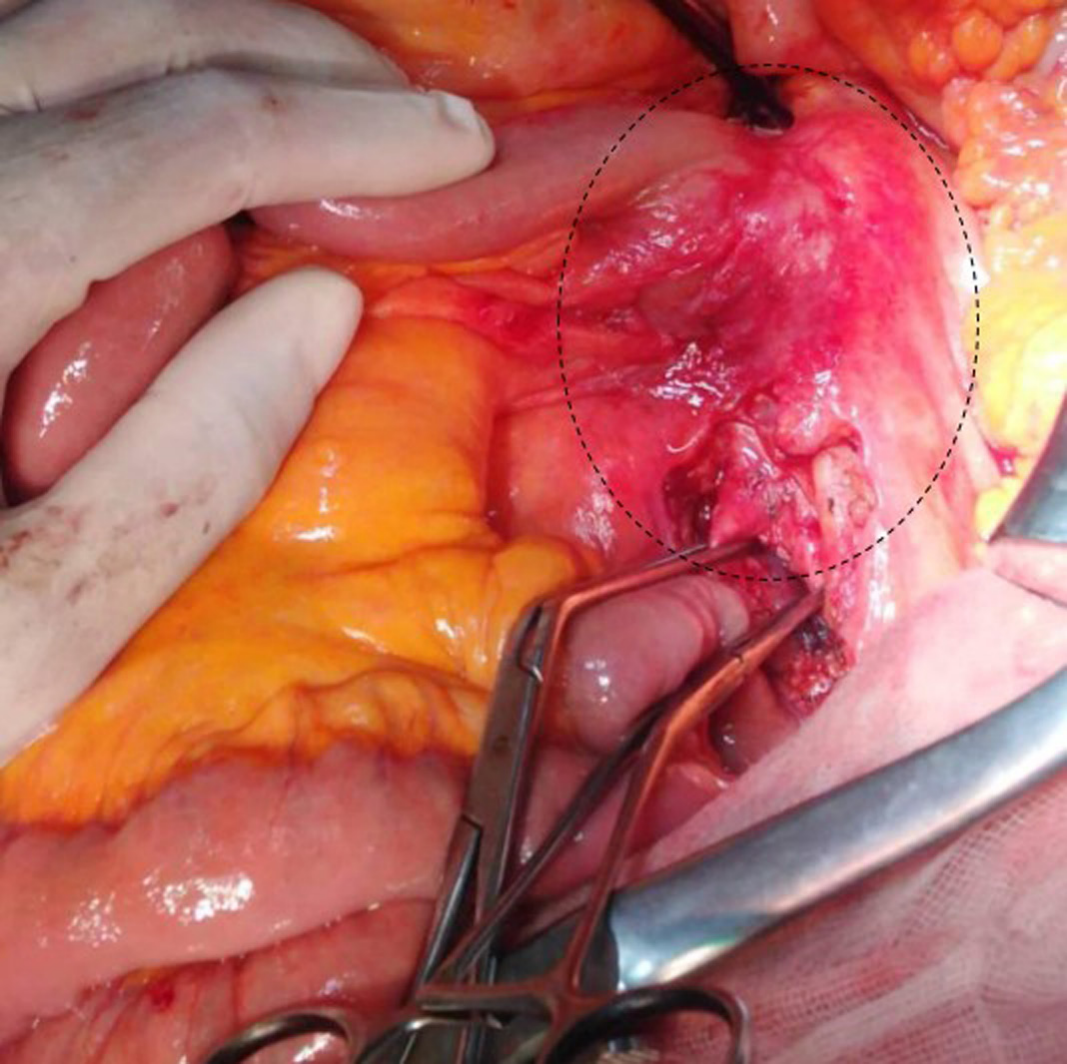
Intraoperative findings of aortoduodenal fistula between the fourth part of the duodenum and the infra‐renal aortic aneurysm (black dotted circle).

**FIGURE 4:**
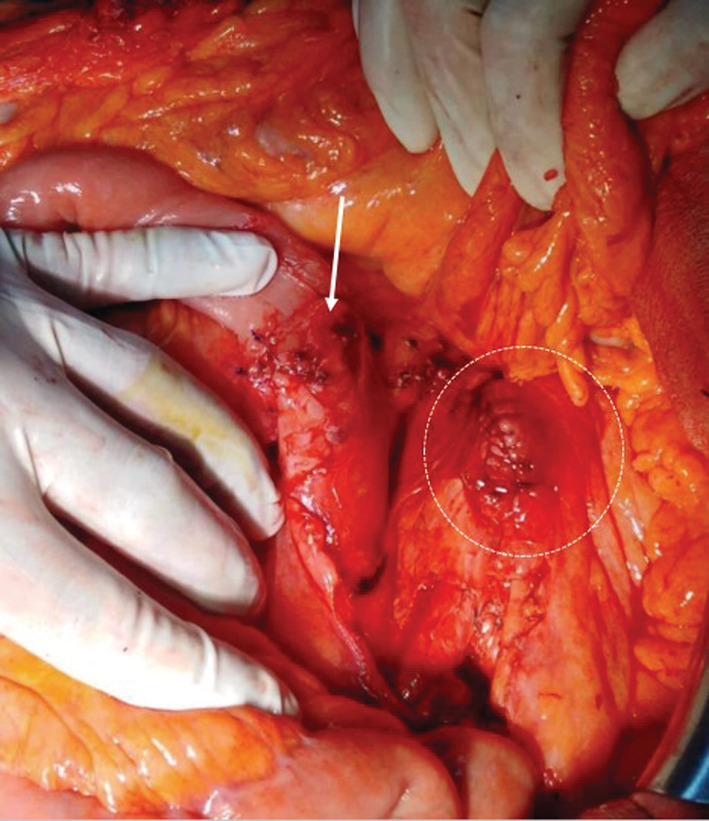
Final look after aortic (white dotted circle) and duodenal repair (white arrow)

A 64‐year‐old man, with a history of hypertension and diabetes, was admitted to our surgical department for acute abdominal pain and massive hematemesis. At the time of admission, his heart rate was at 110 beats/min, his blood pressure was at 90/70 mmHg, and his respiratory rate was at 16 breaths. The hemoglobin level and hematocrit were at 8 g/dl and 35%, respectively. After initial resuscitation, no definite source of the bleeding was found during the upper endoscopy. An emergent abdominal CT scan revealed an infra‐renal abdominal aortic aneurysm measuring 4 cm (Figure 1) with induration around the aorta and contrast leakage toward the duodenum (Figure 2). An immediate laparotomy was performed. During exploration, we identified an aortoduodenal fistula between the fourth part of the duodenum and the proximal infra‐renal aortic aneurysm (Figure 3). There was no evidence of infection. After clamping above and below the aneurysm, the ADF was resected and the aorta was sutured through separate stitches with 4/0 Prolene. The duodenum was closed with a 3/0 Vicryl (Figure 4). The patient had a prolonged postoperative course in the intensive unit. On the 8th day, he suddenly had recurrent hematemesis with severe hemorrhagic shock. The patient was prepared for emergency surgery. Unfortunately, he died in the preoperative stage.

A primary aortoenteric fistula is defined as the spontaneous development of communication between the gastrointestinal tract and the native aorta.^1^

The fistulas most commonly erode into the third part of the duodenum because of the duodenum's close anatomical approximation and fixed nature.^2^ Usually, ADFs are revealed by herald GI bleeding before a severe hemorrhagic shock in the context of a previous aortic aneurysm. The classic triad of GI bleeding, abdominal pain, and pulsating abdominal mass occurs in <25% of patients.^2^, ^3^ Upper endoscopy and CT angiography are helpful to establish the diagnosis.^2^ Primary ADF may be treated with several surgical approaches. Surgery is the treatment of choice to disconnect the vascular tree from the gastrointestinal tract. Ideally, the aorta can be repaired with a simple suture or with graft interposition.^3^ The axillobifemoral bypass is reserved for high‐risk situations (extensive local sepsis or peritonitis).^3^ Nowadays, both extra‐anatomical bypass and closure of the gastrointestinal tract alone are abandoned surgical approaches with poor results. Finally, surgical management of primary ADF is heterogeneous, and there is no agreed consensus with no large or long‐term studies. Endovascular repair is an attractive treatment option and may lead to a revolution in managing ADF.^3^

## AUTHOR CONTRIBUTIONS

Haitham Rejab conceived and designed the study. Haitham Rejab, Sami Fendri, and Ahmed Bouzid were responsible for data collection and acquisition of data. Ahmed Bouzid, Sami Fendri, and Ayman Trigui analyzed and/or interpreted the data. Ahmed Bouzid, Ayman Dammak, and Hela Ben Jemaa wrote the initial manuscript. Haitham Rejab, Ayman Trigui, and Salah Boujelben critically revised the manuscript. All authors have approved the final manuscript.

## CONFLICT OF INTEREST

All authors declare that they had no financial support or relationships that may pose a conflict of interest.

## CONSENT

Written informed consent was obtained from the patient to publish this report by the journal's patient consent policy.

## Data Availability

Data openly available in a public repository that issues datasets with DOIs.
